# Primary Malignant Fibrous Histiocytoma: A Rare Case

**DOI:** 10.1155/2011/134801

**Published:** 2011-10-18

**Authors:** Anastasios Katsourakis, George Noussios, Iosif Hadjis, Neofitos Evangelou, Efthimios Chatzitheoklitos

**Affiliations:** ^1^Department of Surgery, “Agios Dimitrios” General Hospital of Thessaloniki, 54634 Thessaloniki, Greece; ^2^Laboratory of Anatomy, Department of Physical Education and Sports Medicine (at Serres), “Aristotelian” University of Thessaloniki, 62100 Serres, Greece

## Abstract

Malignant fibrous histiocytoma (MFH) of the small intestine is an extremely rare condition. It occurs most commonly in the extremities and the trunk. We report a case of a 67-year-old woman who admitted with fever, myalgia, and altered status. After thorough investigation, a tumor of the jejunum was found. The patient underwent complete surgical removal of the tumor. A diagnosis of MFN (undifferentiated high-grade pleomorphic sarcoma) was made. The patient received adjuvant chemotherapy with Gemcitabine. Two years after the operation, the patient died due to recurrence of the disease. MFH of the small intestine is an extremely rare neoplasm with an aggressive biological behaviour. In this paper, pathogenesis, natural history, and treatment are reviewed.

## 1. Introduction

Malignant fibrous histiocytoma is a soft-tissue tumor sarcoma of mesenchymal origin. The site of primary origin tends to be mainly in the extremities followed by the trunk, the head, and the neck. It is the most common soft-tissue sarcoma with the peak incidence in the seventh decade. Although MFH is the most common soft-tissue sarcoma in late adult life, intestinal involvement has rarely been reported. A review of the literature revealed 41 cases. This report describes a case of MFH arising in the small intestine [1, 2].

## 2. Case Report

A 67-year-old woman was admitted to the department of internal medicine due to persistent fever (39°C max), weight loss, poor appetite, myalgia, and fatigue. Personal history of the patient revealed total hysterectomy 28 years ago and radiotherapy due to endometrial cancer. 

**Figure 1:**
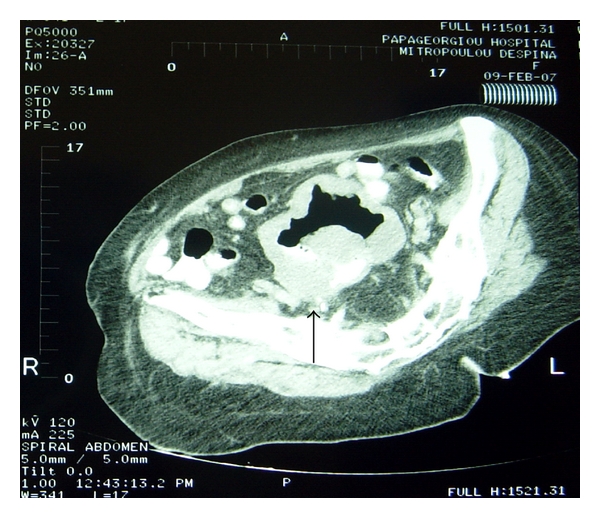
Preoperative CT examination of the patient.

Physical examination on admission showed slight abdominal distension without tenderness and no mass palpable. Laboratory examination showed 11,400 WBC with normal differential count. Total protein level was normal (7.2 U/L), but the globulin level was slightly elevated (3.94 mg/dL). Tumor markers (CEA, Ca 19-9, Ca 125, CA 15-3, alpha-foetoprotein) were within normal values. Ultrasonography of the abdomen revealed a mass at the left lower abdominal cavity. Computed tomography of the thorax was normal, while the one of the abdomen and the retroperitoneal space revealed a tumor within the lesser pelvic cavity in the proximity of the small intestine (Figure 1).

**Figure 2:**
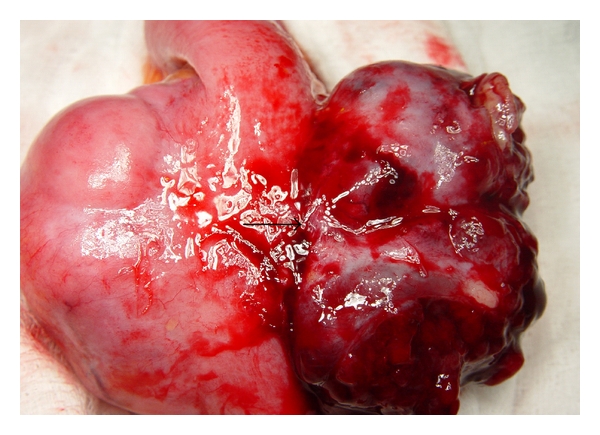
Intraoperative finding.

**Figure 3:**
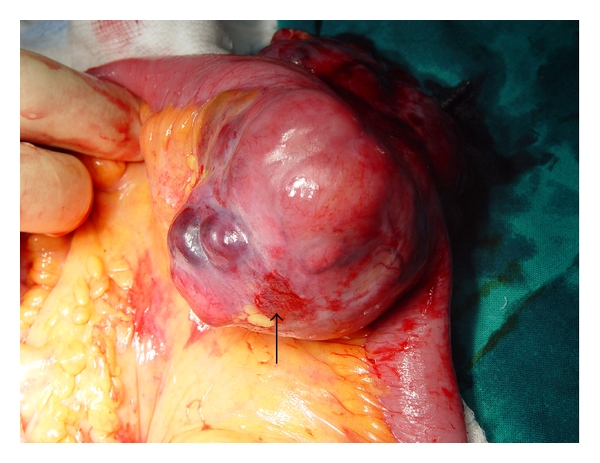
Intraoperative finding.

At surgery, we found a tumor mass originating from the wall of the small intestine (jejunum), invading the mesentery (Figures 2 and 3). There was no sign of intraabdominal spread, and wide resection of the tumor with intestinal side to side anastomosis was performed. 

**Figure 4:**
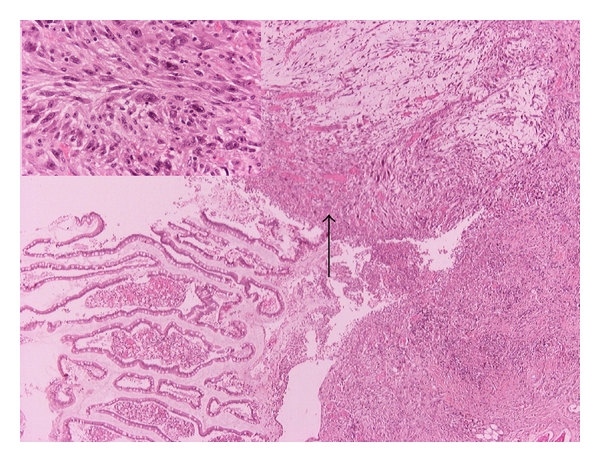
Microscopy with H and E staining ×40, left up ×400.

**Figure 5:**
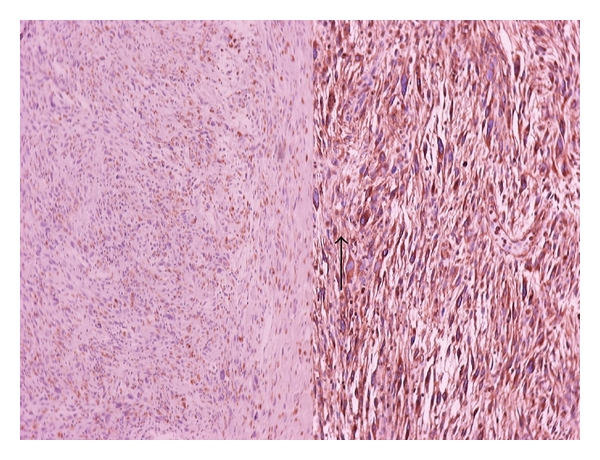
Immunohistochemistry with vimentin (left) and CD 68/100 (right).

The tumor measured 6, 4 × 4 × 4, 5 cm, and the cut surface of the tumor was whitish-brown in color and had a solid appearance. Microscopically, the tumor mass had a submucosal location, and it had invaded the muscular layer with no signs of serosal, perineural, or vascular invasion (Figure 4). The histopathological examination demonstrated a storiform pattern of growth with lymphocytic and neutrophilic infiltrates and dispersed atypical, spindle- or oval-shaped cells. Pleomorphic mono-, multinucleated cells with bizarre nuclei were also intermingled in the lesion. Mitotic figures were pronounced immunohistochemically, and the tumor cells were positive to vimentin and CD-68 antigen but negative to desmin, S100 protein, cytokeratins AE1/AE3, CD117, and CD34 antigen (Figure 5).

Pathology diagnosis was storiform/pleomorphic MFH (current WHO classification: undifferentiated high-grade pleomorphic sarcoma).

The postoperative period was uneventful, and the patient was discharged one week after the operation. The patient received adjuvant chemotherapy based on Gemcitabine. Unfortunately, two years after the operation, she suffered from recurrence of the tumor with lung metastasis and died.

## 3. Discussion

Malignant soft-tissue tumors of the small intestine are extremely rare. The most common type is leiomysarcoma [3]. MFH was first described as malignant histiocytoma and fibrous xanthoma by Ozello et al. in 1963 and was established by O'Brien and Stout in 1964 to describe soft-tissue sarcomas arising from fibroblasts and histiocytes [4, 5]. MFH has varied histology morphology, but the classic form is composed of spindle-shaped and round histiocytes arranged in storiform pattern and accompanied by inflammatory cells as in our case.

MFH is considered to be a rare malignancy of visceral organs. It has been described in the lung, kidney, liver, stomach, duodenum, pancreas, colon, and anal canal. It usually occurs in the extremities, presenting as a painless mass, and less commonly in the retroperitoneal space, associated with weight loss and increased intra-abdominal pressure [6]. Five histological subtypes of MFH have been described: pleomorphic storiform and myxoid (most common types), giant cell, inflammatory, and angiomatoid [7].

The karyotypic abnormalities in MFH are usually complex, with multiple numerical and structural rearrangements. Schmidt reported that chromosomes 1, 3, 6, 9, 12, 16, 18, and 20 are involved in structural aberrations and that the breakpoint regions are most frequently observed in 1p32, 3p25, and in the centromeric region of chromosomes 1 and 16. The pathogenesis of MFH has not been clarified to date.

However, it has been recognized as a complication of radiation, resulting from chronic postoperative repair, trauma, surgical incisions, or burn scars [8, 9].

The diagnosis of MFH depends on an accurate differential diagnosis from other sarcomas, observation of karyomorphism and differential figures, and positive results on immunohistological staining. It was reported that MFH frequently expresses vimentin, actin, CD-68, and *α* 1-antitrypsin and *α* 1-antichymotrypsin. The differential diagnosis of MFH should include pleomorphic liposarcoma and rhabdomyosarcoma. The former lacks the storiform pattern and shows evidence of cellular differentiation, while the latter shows cross striations on histological examination [10]. 

Liesveld et al. reported that patients with MFH have leukocytosis, leukemoid reaction, and paraneoplastic syndrome because of various cytokines produced by tumor cells. Thus, postoperative recurrent leukocytosis and elevated CRP level might be predictors for recurrence of MFH [11]. 

The biological behaviour of malignant fibrous histiocytoma is extremely aggressive, and the prognosis is presumably poor, mainly depending on the size and histological grading.

The treatment for MFH is early and complete surgical excision with en-bloc regional lymph node dissection. Chemotherapy (Doxorubicin or Gemcitabine or combination of Doxorubicin and Decarbazine, and Doxorubicin, Mesna, and Ifosfamide) or radiation is recommended in those patients in whom there is vascular or lymphatic infiltration. Zagars et al. reported that adjuvant chemotherapy cannot minimize the rate of metastasis [12]. Patients with myxoid tumors do not require systemic therapy. However, patients with nonmyxoid disease exceeding 5 cm are at a significant risk of developing metastases, and the development of effective adjuvant treatment is an important research goal [12]. Most of the reports suggest that the prognosis associated with colonic MFH is poor. Weiss and Enzinger's analysis of MFH showed that the 2-year survival rate of patients with pleomorphic/storiform type of MFH is 60% and the rate of metastases is 42% [6].

In conclusion, primary intestinal histiocytoma is an extremely rare neoplasm with an aggressive biological behavior. Complete surgical resection is preferred, and adjuvant chemotherapy or radiotherapy may be advisable. Due to the recurrence, lifelong surveillance should be carried out.

## References

[1] Okubo H, Ozeki K, Tanaka T, Matsuo T, Mochinaga N (2005). Primary malignant fibrous histiocytoma of the ascending colon: report of a case. *Surgery Today*.

[2] Fu DL, Yang F, Maskay A (2007). Primary intestinal malignant fibrous histiocytoma: two case reports. *World Journal of Gastroenterology*.

[3] Hasegawa S, Kawachi H, Kurosawa H (2004). Malignant fibrous histiocytoma in the ileum associated with intussusception. *Digestive Diseases and Sciences*.

[4] Ozzello L, Stout AP, Murray MR (1963). Cultural characteristics of malignant histiocytomas and fibrous xanthomas. *Cancer*.

[5] O'Brien JE, Stout AP (1964). Malignant fibrous xanthomas. *Cancer*.

[6] Weiss SW, Enzinger FM (1978). Malignant fibrous histiocytoma: an analysis of 200 cases. *Cancer*.

[7] Anagnostopoulos G, Sakorafas GH, Grigoriadis K, Kostopoulos P (2005). Malignant fibrous histiocytoma of the liver: a case report and review of the literature. *Mount Sinai Journal of Medicine*.

[8] Froehner M, Gaertner HJ, Hakenberg OW, Wirth MP (2001). Malignant fibrous histiocytoma of the ileum at a site of previous surgery: report of a case. *Surgery Today*.

[9] Kim GI, Lee JH, Kim HK, Park SH, Kim CH (2004). Malignant fibrous histiocytoma in a chronic burn scar: a rare case report and review of the literature. *Burns*.

[10] Rosenberg AE (2003). Malignant fibrous histiocytoma: past, present, and future. *Skeletal Radiology*.

[11] Liesveld JL, Rush S, Kempski MC (1993). Phenotypic characterization of the human fibrous histiocytoma giant cell tumor (GCT) cell line and its cytokine repertoire. *Experimental Hematology*.

[12] Zagars GK, Mullen JR, Pollack A (1996). Malignant fibrous histiocytoma: outcome and prognostic factors following conservation surgery and radiotherapy. *International Journal of Radiation Oncology Biology Physics*.

